# Supplementary material for: Intake Estimation of Phytochemicals in a French Well-Balanced Diet
Source: Nutrients. 2021 Oct 16;13(10):3628. doi: 10.3390/nu13103628 (PMC8539512; doi:10.3390/nu13103628)
Supplement: Supplementary file 1 [file nutrients-13-03628-s001.zip › nutrients-1424911-SI.pdf]

Target values of phytonutrients in a healthy diet:

perspectives for updating dietary recommendations in France

Authors

Marie-Josèphe Amiot,[1] Christian Latgé,[2] Laurence Plumey,[3] Sylvie Raynal[4]

Affiliations

1 INRAE, MoISA, University of Montpellier, CIHEAM-IAMM, CIRAD, INRAE, Institut Agro, IRD, 2 place Pierre Viala, 34000 Montpellier, France

2 Pierre Fabre Laboratories, 5 Rue Saint-Pantaléon, 31000 Toulouse, France

3 EPM Nutrition School, 11 avenue des Vignes, 92210 St Cloud, France

4 Naturactive, Pierre Fabre Laboratories, 29 avenue du Sidobre, 81106 Castres, France

Corresponding author

Marie-Josèphe Amiot

MoISA

University of Montpellier

2 place Pierre Viala

34000 Montpellier

France

Email: marie-josephe.amiot-carlin@inrae.fr

Telephone: +33 (0)4 99 61 22 16

Supplementary Materials

Table S1. Summer menu

Day	Breakfast	Lunch	Dinner	No. of portions
**Monday**	Tea<br><br>Two slices of wholemeal bread with honey (= 1 carbohydrate)<br><br>One peach	Tomato salad (= 1 tomato)<br><br>Moussaka (= 2 aubergines)<br><br>Strawberries (1 portion)	Green salad (= 0.5 portions)<br><br>Wild rice with vegetables (= 1 wholegrain rice + 1 red pepper)<br><br>Apricot compote<br><br>Red wine (1 glass)	Herbs: 1<br>Carbohydrates: 2<br>Orange: 2<br>Red: 3<br>Tea: 1<br>Green: 0.5<br>Red wine: 1<br>Purple: 2
**Tuesday**	Coffee<br><br>Two slices of wholemeal bread with honey (= 1 carbohydrate)<br><br>One apricot	Asparagus with vinaigrette (= 1 portion)<br><br>Sautéed runner beans and cauliflower (= 1 runner beans + 1 cauliflower + 0.5 shallot)<br><br>Half a grapefruit (= 1 portion)	Chick pea stew with tomatoes and onions (= 1 portion of pulses + 1 tomato + 0.5 onion)<br><br>One slice of bread<br><br>One banana	Garlic and onion: 0.5<br>Herbs: 1<br>White: 1<br>Filter coffee: 1<br>Carbohydrates: 2<br>Yellow: 1<br>Orange: 1<br>Pulses: 1<br>Red: 2.5<br>Green: 2

Day	Breakfast	Lunch	Dinner	No. of portions
**Wednesday**	Tea<br><br>Two slices of wholemeal bread with honey (= 1 carbohydrate)<br><br>One apricot	Greek-style mushrooms (= 1 portion)<br><br>Braised fennel (= 2 portions)<br><br>Blackberries (1 portion)	Lamb's lettuce salad (= 0.5 portions)<br><br>Green beans (= 2 portions)<br><br>One slice of bread<br><br>One fig	Herbs: 1<br>White: 1<br>White/green: 2<br>Carbohydrates: 2<br>Orange: 1<br>Red: 1<br>Tea: 1<br>Green: 2.5<br>Purple: 1
**Thursday**	Coffee<br><br>Two slices of wholemeal bread with honey (= 1 carbohydrate)<br><br>One nectarine	Cucumber with vinaigrette (= 1 portion)<br><br>Courgettes with parsley (= 2 portions)<br><br>Blackcurrants (1 portion)	Cod en papillote with carrots and olive oil (= 2 portions)<br><br>One slice of bread<br><br>One peach	Herbs: 2<br>Filter coffee: 1<br>Carbohydrates: 2<br>Orange: 2<br>Orange vegetable: 1<br>Green: 2<br>Purple: 1
**Friday**	Tea<br><br>Two slices of wholemeal bread with honey (= 1 carbohydrate)<br><br>Melon (1 portion)	Grated carrots with vinaigrette (= 1 portion carrots)<br><br>Oven-baked mackerel with tomatoes, onions, white beans, white wine (= 1 pulses + 1 tomato + 0.5 onion)<br><br>Half a grapefruit (= 1 portion)	Lentil tabbouleh with vegetables (= 1 portion of lentils + 1 radish + 1 yellow pepper + 0.5 onion)<br><br>One slice of bread<br><br>Pineapple (1 portion)<br><br>Red wine (1 glass)	Garlic and onion: 0.5<br>Herbs: 1<br>Carbohydrates: 3<br>Yellow: 2<br>Orange: 1<br>Orange vegetable: 1<br>Pulses: 1<br>Radish: 1<br>Red: 2<br>Tea: 1<br>Red wine: 1

Day	Breakfast	Lunch	Dinner	No. of portions
**Saturday**	Two slices of wholemeal bread with honey (= 1 carbohydrate)<br><br>Raspberries (1 portion)	Tomato salad<br><br>Sautéed potatoes (= 1 carbohydrate)<br><br>Cherries (1 portion)	Warm goat's cheese salad with nuts and bacon lardons (= 1 portion salad + 1 tomato + 0.5 onion<br><br>One slice of bread<br><br>Peach compote	Garlic and onion: 0.5<br>Herbs: 2<br>Carbohydrates: 3<br>Orange: 1<br>Red: 3<br>Green: 1
**Sunday**	Tea<br><br>Two slices of wholemeal bread with honey (= 1 carbohydrate)<br><br>One fig	Black radish salad (= 1 black radish)<br><br>Green beans with parsley (= 2 portions)<br><br>Strawberries (1 portion)	Wild rice (= 1 portion of wholegrain rice)<br><br>Tuna with tomato sauce (= 0.5 tomato + 0.5 onion)<br><br>Blueberries (1 portion)	Garlic and onion: 0.5<br>Herbs: 2<br>Carbohydrates: 2<br>Radish: 1<br>Red: 1.5<br>Tea: 1<br>Green: 2<br>Red wine: 1<br>Purple: 1

Table S2. Winter menu

Day	Breakfast	Lunch	Dinner	No. of portions
**Monday**	Tea<br><br>Two slices of wholemeal bread with honey (= 1 carbohydrate)<br><br>One orange	Chick pea salad<br><br>Sweet potato parmentier (= 2 portions of sweet potato)<br><br>One kiwifruit	Green salad (= 0.5 portions of green salad leaves)<br><br>Wild rice with vegetables (= 1 wholegrain rice + 1 leek)<br><br>Sugar-free compote (= 1 apple)	Herbs: 1<br>White: 1<br>White/green: 1<br>Carbohydrates: 3<br>Yellow/orange: 1<br>Orange vegetable: 2<br>Tea: 1<br>Green: 1.5
**Tuesday**	Coffee<br><br>Two slices of wholemeal bread with honey (= 1 carbohydrate)<br><br>One apple	Chicory with vinaigrette (= 1 chicory)<br><br>One slice of bread<br><br>Salsify (= 2 portions)<br><br>Half a grapefruit (= 1 portion)	Chick pea stew with carrot (= 1 carbohydrate + 1 carrot + 0.5 onion)<br><br>One orange	Garlic and onion: 0.5<br>Herbs: 1<br>White: 4<br>Filter coffee: 1<br>Carbohydrates: 2<br>Yellow/orange: 1<br>Orange vegetable: 1<br>Pulses: 1<br>Red: 1
**Wednesday**	Tea<br><br>Two slices of wholemeal bread with honey (= 1 carbohydrate)<br><br>One pear	Beetroot with vinaigrette (= 1 portion)<br><br>One slice of bread<br><br>Cauliflower (= 1 portion)<br><br>One apple	Lamb's lettuce salad (= 0.5 portion)<br><br>Squash gratin (= 2 portions)<br><br>One banana	Herbs: 1<br>White: 2<br>Carbohydrates: 2<br>Yellow: 1<br>Orange vegetable: 2<br>Red: 1<br>Tea: 1<br>Green: 0.5<br>Cabbage: 1

Day	Breakfast	Lunch	Dinner	No. of portions
**Thursday**	Coffee<br><br>Two slices of wholemeal bread with honey (= 1 carbohydrate)<br><br>One persimmon	Greek-style mushrooms (= 1 portion)<br><br>White beans with spinach (= 1 portion of greens + 1 portion of pulses)<br><br>One pear	Cod en papillote with celery and olive oil (= 2 portions celery)<br><br>One slice of bread<br><br>One orange	Herbs: 2<br>White: 2<br>White/green: 2<br>Filter coffee: 1<br>Carbohydrates: 2<br>Yellow/orange: 1<br>Orange: 1<br>Green: 2
**Friday**	Tea<br><br>Two slices of wholemeal bread with honey (= 1 carbohydrate)<br><br>Red grapes (1 portion)	Rocket salad (= 0.5 portion)<br><br>Steamed broccoli with onions and potatoes (= 1 portion carbohydrates + 0.5 onion<br><br>Half a grapefruit (= 1 portion)	Red bean soup with onions and sweetcorn (= 1 portion of pulses + 1 portion of sweetcorn + 0.5 onion)<br><br>Baked apple (= 1 apple)<br><br>Red wine (1 glass)	Garlic and onion: 1<br>Herbs: 1<br>White: 1<br>Carbohydrates: 3<br>Pulses: 1<br>Red: 1<br>Tea: 1<br>Green: 0.5<br>Red wine: 1<br>Purple: 1
**Saturday**	Two slices of wholemeal bread with honey (= 1 carbohydrate)<br><br>One kiwi	White cabbage salad (= 1 portion)<br><br>One slice of bread<br><br>Sautéed potatoes (= 1 carbohydrate)<br><br>One apple	Warm goat's cheese salad with sesame seeds and bacon lardons (= 1 portion of salad, 1 portion of sesame seeds + 0.5 onion)<br><br>Quince compote (= 1 quince)	Garlic and onion: 0.5<br>Herbs: 2<br>White: 2<br>Carbohydrates: 3<br>Oil: 1<br>Green: 1<br>Cabbage: 1

Day	Breakfast	Lunch	Dinner	No. of portions
**Sunday**	Tea<br><br>Two slices of wholemeal bread with honey (= 1 carbohydrate)<br><br>One mandarin	Red chicory salad (= 1 portion)<br><br>Peas (= 2 portions)<br><br>Red grapes (1 portion)	Wild rice (= 1 wholegrain rice)<br><br>Jerusalem artichoke (= 1 portion)<br><br>One pear<br><br>Red wine (1 glass)	Herbs: 1<br>White: 1<br>Carbohydrates: 3<br>Yellow/orange: 1<br>Tea: 1<br>Green: 2<br>Red wine: 1<br>Purple: 2
